# Review of Veterinary Sanitary Measures

**Published:** 1895-03

**Authors:** 


### REVIEW OF VETERINARY SANITARY MEASURES.

The Live Stock Commission of Maine makes the following comment upon the increase of glanders among horses of that State: In 1893 but 22 were found infected, which were condemned and destroyed at an appraisal of $995. In 1894, 64 have been condemned at an appraisal of $2692.50, and the average number destroyed for the past five years is less than 15, and up to a year ago their report indicated that but a very limited number of horses destroyed were bred in Maine, the great majority of these being Western horses. Maine, in 1888, quarantined against Texas mustangs and bronco horses, which were sold in that State to the number of 2000 in 1887, among which 13 cases of glanders were found in a single drove. Since that time no more bronchos have been allowed to be brought into the State, but a very great many loads of Western horses of a cheaper grade have been sold there, many of which have been exposed to glanders before coming into the State. The Commission advises some additional restraint on the introduction of these horses, but do not think the Western quarantine justifiable, as it would militate against the interests of very many honorable dealers in different parts of Maine who deal in work and business horses, and for which the demand seems to exceed the supply of Maine-bred horses.

---

The class officers of the American Veterinary College for the year 1895 are as follows: *President*, Thomas Smith; *Vice President*, Augustus Blake; *Secretary*, A. Mitchell; *Treasurer*, William Laws; *Historian*, Martin J. Dair.

Professor Osler, of Johns Hopkins University, at a recent meeting of the Philadelphia Alumni of the University of Pennsylvania, speaking on the subject of "Lines of Future Development in Medical Education," advocated eliminating from the medical course the unmedical subjects, such as general chemistry, biology, and physics. He made a strong plea for a more practical education for students, more bedside training in the hospital wards. His views in regard to more training and practical experience might be well taken by some of our veterinary colleges.

A useful and effective febrifuge mixture in influenza and catarrhal fevers in horses:

℞—Tinc. Belladonnæ . . . . f.℥iss

Tinc. Cinchonæ Rubri Comp. . . f.℥iiss

Sp. Æth. Nitrosi

Liq. Ammon. Acetas . . . āā f.℥iv. M.

S.—Give one ounce every two hours on the tongue with a syringe.

A veterinarian resident of Western Pennsylvania, who had recently passed the civil service examination and received an appointment to a place in the Bureau of Animal Industry, has had his appointment revoked on the grounds that during the campaign of 1892 his partisan zeal had seemed to get the better of his judgment, and leading to the commission of such grave acts of offensive partisanship, for which he is now made to suffer.

